# Requirements for market entry of gene drive-modified mosquitoes for control of vector-borne diseases: analogies to other biologic and biotechnology products

**DOI:** 10.3389/fbioe.2023.1205865

**Published:** 2023-06-08

**Authors:** Stephanie L. James, Hector Quemada, Mark Q. Benedict, Brinda Dass

**Affiliations:** ^1^ GeneConvene Global Collaborative, Foundation for the NIH, Bethesda, MD, United States; ^2^ Independent Consultant, Kalamazoo, MI, United States; ^3^ CDC Foundation, Atlanta, GA, United States

**Keywords:** malaria, mosquito, genetic modification, gene drive, market entry, regulatory approval, supply chain, manufacturing standards

## Abstract

Gene drive-modified mosquitoes (GDMMs) are proposed as new tools for control and elimination of malaria and other mosquito-borne diseases, and promising results have been observed from testing conducted in containment. Although still at an early stage of development, it is important to begin now to consider approval procedures and market entry strategies for the eventual implementation of GDMMs in the context of disease control programs, as these could impact future research plans. It is expected that, as for other types of new products, those seeking to bring GDMMs to market will be required to provide sufficient information to allow the regulator(s) to determine whether the product is safe and effective for its proposed use. There already has been much emphasis on developing requirements for the biosafety components of the “safe and effective” benchmark, largely concerned with their regulation as genetically modified organisms. Other potential approval requirements have received little attention, however. Although GDMMs are expected to be implemented primarily in the context of public health programs, any regulatory analogies to other public health products, such as pharmaceuticals, vaccines, or chemical pesticides, must take into account the characteristics of live mosquito products. Typical manufacturing standards related to product identity, potency or quality will need to be adapted to GDMMs. Valuable lessons can be drawn from the regulatory approval processes for other whole organism and genetically modified (GM) organism products. Supply chain requirements, such as scale of production, location and design of production facilities, and methods of distribution and delivery, will be dependent upon the characteristics of the particular GDMM product, the conditions of use, and the region to be served. Plans for fulfilling supply chain needs can build upon experience in the development of other live insect products for use in public health and agriculture. Implementation of GDMMs would benefit from additional research on enabling technologies for long-term storage of mosquito life stages, efficient mass production, and area-wide delivery of GDMMs. Early consideration of these practical requirements for market entry will help to mitigate downstream delays in the development of these promising new technologies.

## Introduction

The World Health Organization (WHO) estimates that over 700,000 deaths occur annually from parasitic, bacterial, and viral diseases transmitted by insect or other invertebrate vectors (World Health Organization, 2023a). Malaria alone, a parasitic disease transmitted by anopheline mosquitoes, was reported to cause some 619,000 deaths worldwide in 2021, approximately 96% of which occurred in Africa (World Health Organization, 2022a). There have been multiple calls to improve methods for malaria treatment and prevention (e.g., Rabinovich et al., 2017; Feachem et al., 2019; World Health Organization, 2019). These include calls for innovative vector control tools.

Gene drive-modified mosquitoes (GDMMs) have been recognized as potentially transformative new tools for control and elimination of malaria and other mosquito-borne diseases (World Health Organization, 2020a). Although other species also transmit malaria in Africa, gene drive research currently is most advanced in mosquitoes of the *Anopheles gambiae* species complex, which historically have been important malaria vectors in that region (Sinka, 2012; Global Health Network, 2023).

WHO guidance on the research and development pathway for GDMMs (World Health Organization, 2021) calls for testing initially to be conducted under physical confinement, as in insectaries or large cages. The WHO guidance recommends that during early confined testing measurable efficacy and biosafety surrogate indicators should be identified that can be expected to correlate with the ability of the GDMM product to accomplish the intended use in the field (the product claim). Reports of success already are coming from such contained testing, with results based on defined endpoints supporting the investigational claim (Kyrou et al., 2018; Pham et al., 2019; Carballar-Lejarazu et al., 2020; Hammond et al., 2021; Ellis et al., 2022). The identified efficacy and safety correlates, as well as the intended use, will be captured in a product-specific Target Product Profile, and will inform the proposed testing endpoints in regulatory applications submitted by the developer and evaluated by national regulators as the basis for advancing through open field releases of increasing size and scope. For products making a disease reduction claim, field releases eventually would encompass a large enough area to allow for assessment of any resulting reduction in malaria transmission (World Health Organization, 2021). Efficacy and safety data and information from appropriately designed field trials will provide the scientific evidence supporting the product claim and intended use as a vector control and/or malaria control tool in an application for approval for market entry, which will be evaluated by the appropriate regulatory authorities. GDMM products making a vector control claim could be regulated differently from those making a public health claim (James et al., 2023). Market entry is defined here to mean that the product has undergone regulatory approval and is being made available to end users, whether in return for payment or free of charge. No form of GDMMs has yet progressed to field testing. Nevertheless, in order to plan for success it is not too early to begin considering the needs for bringing GDMMs to market as public health tools.

### Implementation considerations

The GDMM product previously has been defined as any life stage of the transgenic mosquitoes that is produced under controlled conditions for deliberate release (James et al., 2018; James et al., 2020; James et al., 2023). If approved for market entry, those responsible for national or regional disease control priorities will decide whether the GDMM product should move into wider and more systematic releases as part of a national or regional malaria control program. The implementation phase is the post-investigational use of GDMMs, following satisfactory demonstration of safety, efficacy and acceptability in field trials and a decision to initiate widescale releases (World Health Organization, 2021). This phase can be considered analogous to commercialization of more familiar biotechnology and public health products such as drugs, vaccines, insecticides, and crops. Commercialization is broadly defined as a process for bringing new products or services to market (as defined above). The scope of the commercialization process generally includes regulatory approval (including fulfillment of agreed-upon post-approval requirements), production, distribution, marketing, and other key functions that will be as critical for success of a GDMM product as they are for other products. Yet these practical aspects of operationalizing GDMMS have received little attention to date.

Rearing of GDMMs shares many characteristics with manufacturing of other types of public health products. Implementation of GDMMs will require provision of a consistent product at the necessary scale, as well as its delivery in a way that is designed to reliably achieve the claimed vector control or other public health effect. Plans for achieving area-wide protection by GDMMs are likely to be more context specific than is the case for medical or public health products aimed at individual or household use. Thus, specifics of a release plan, including the location of release sites, how many GDMMs will be released at each site, and how often these releases will occur at each site, may differ for each product in a particular setting. This will depend upon factors such as the type of gene drive system and heritability of the transgenic construct (Box 1), the population size of the targeted mosquito species at the site, and biological traits of the released male GDMMs such as mating competitiveness with respect to wild males (e.g., North et al., 2019; Kaiser et al., 2021). In general, self-sustaining gene drive products are expected to require release of lower numbers of GDMMs over a shorter period of time to yield a long-term effect. Because they are expected to require relatively larger and/or more frequent releases to sustain effectiveness in the region of interest, self-limiting GDMM approaches are likely to require greater capacity for production and delivery. Moreover, this level of production and delivery may need to be maintained long-term, since it has been found with other genetic biocontrol methods that re-invasion by the targeted species can occur rapidly following cessation of the control measure (e.g., Meyer et al., 2016). Localizing GDMMs are likely to require more extensive releases to provide widespread coverage, which also will have production and delivery ramifications.

BOX 1 **A primer on gene drive systems**

Genetically modified (GM) mosquitoes (also called genetically engineered, transgenic, or living modified mosquitoes) demonstrate traits that are introduced through use of recombinant DNA technology. Gene drive refers to a process, either naturally occurring or resulting through use of recombinant DNA technology, whereby a particular gene or genetic construct is able to enhance its own inheritance so that it becomes more prevalent in the population over successive generations (Alphey et al., 2020). Engineered gene drive systems can be used to introduce new and potentially beneficial traits rapidly into a population. Gene drive-modified mosquitoes (GDMMs) are a subset of GM mosquitoes that contain an engineered gene drive system.

Several different gene drive systems have been proposed for use in preventing transmission of mosquito-borne diseases, and others likely will be developed in the future (reviewed in World Health Organization, 2021). These systems currently aim either to reduce the size of the vector population by inhibiting their reproduction or survival (a strategy termed population suppression or reduction) or to modify the mosquitoes to make them less competent to transmit a pathogen (variously termed population replacement, modification, conversion, or alteration). Self-sustaining drives are intended to persist, passing the modification on through subsequent generations indefinitely. Because of this persistence, many self-sustaining drives are expected to spread widely within interbreeding mosquito populations. Low threshold drives are a type of self-sustaining system in which this spread can be initiated by release of relatively few modified mosquitoes. Other types of gene drive systems aim to impose either temporal (self-limiting drives) or spatial (localizing or confined drives) restrictions on the spread of the modification. Self-limiting drives will eventually disappear from the target mosquito population, and may effectively be localizing because they do not persist long enough to spread widely. Localizing systems may be either self-limiting or self-sustaining.

Certain challenges are anticipated for large scale production and delivery as required for implementation in the context of national or regional disease control programs. Basic elements of the supply chain for conventional public health products such as drugs, pesticides and vaccines, from production through distribution, are generally established through commercial manufacturers and national health authorities or private sector vendors. In these cases, supply chain experience already exists and the requirements have been thoroughly studied, even though all elements may not be in place for a new product (Brown and Bollyky, 2021) and the supply chain may be fragmentary in inadequately resourced regions (U.S. Agency for International Development, 2011; Yadav, 2015). At least initially, GDMMs may face these same hurdles, as well as additional challenges arising from lack of understanding, experience, or pre-existing infrastructure for this new product class. For example, commercial entities and national health authorities have limited experience with live mosquito products, many current GDMM developers are laboratorians without substantial product development expertise, and regulatory frameworks for GM insects have not been clarified in many countries. Thus, early planning would greatly ease the process of market entry of GDMMs and would best begin in time to allow these challenges to be sufficiently addressed. Regulatory and policy considerations for GDMMs have been detailed elsewhere (e.g., World Health Organization, 2021; James et al., 2023). Here we consider manufacturing standards, production requirements, and delivery mechanisms for GDMMs, and highlight issues that require particular attention to inform planning for commercialization and incorporation into national malaria control programs. Post-implementation monitoring procedures are not considered within the scope of this analysis.

## Manufacturing standards

For public health products such as pharmaceuticals that will be administered to individuals, those seeking to bring new products to market are required to provide sufficient information to allow the regulator(s) to determine whether the product is both safe and effective for the proposed health condition (the product claim) when administered according to the specified conditions of use, and whether the manufacturing, packaging, and storage methods will be able to maintain the integrity of the product across different release lots. These requirements generally involve specifications for product identity, potency, purity, and quality (e.g., U.S. Food and Drug Administration, 1999; European Medicines Agency, 1999; Code of Federal Regulations, 2023). For conventional drugs, assessment typically focuses on analysis of chemical composition, including concentration and stability of the active ingredient and presence of any contaminating components. Registration considerations for pesticides are largely risk-based, but like medicines also require description of all chemicals in the product and proof that the manufacturing process is reliable (e.g., U.S. Environmental Protection Agency, 2023). For biological products such as cell-derived or cell-based therapies, which are manufactured via serial passage and whose active ingredients cannot be straightforwardly chemically characterized, more appropriate types of tests have been developed to confirm that the product meets the claimed identity, strength and quality characteristics (e.g., Rayment and Williams, 2010; Carman et al., 2012). This provides a good example of how manufacturing standards can be adapted to the characteristics of new types of products. GDMMs will be whole organism products. Thus, regulatory approval requirements for products that also are live organisms, including other insect products or GM plants and animals, provide particularly relevant precedents for manufacturing standards for GDMMs intended as public health products (Romeis et al., 2020). However, these requirements may not be as familiar to health regulators.

Requirements for the biosafety components of the “safe and effective” benchmark for GDMMs are actively being addressed. Considerations for safety testing of other GM organisms have been a topic of extensive discussion, both from a human and animal (food) safety as well as an environmental safety perspective (e.g., Codex Alimentarius, 2003; U.S. Food and Drug Administration, 1997; Convention on Biological Diversity, 2000; European Food Safety Authority, 2010; European Food Safety Authority, 2011; European Food Safety Authority, 2013; Organization for Economic Cooperation and Development, 2023). These discussions also have extended to GDMMs and are ongoing (e.g., Convention on Biological Diversity, 2020; European Food Safety Authority, 2020; World Health Organization, 2021). Therefore, we focus here on other standards typically addressed in regulatory approval procedures that have not been similarly examined. These standards are concerned with product quality and consistency rather than safety *per se*, and could involve regulatory agencies other than those responsible for safety assessments (James et al., 2023).

Lessons learned from other live mosquito products that have obtained regulatory approval for large-scale deployment can be especially informative for all aspects of market entry for GDMMs. Perhaps the most well-documented precedent for standardizing production of GDMMs would be practices common to live insect products used in the Sterile Insect Technique (SIT) method for population suppression. SIT has been widely and successfully used to control a variety of agricultural pest insects and is being adapted to mosquitoes. It involves the release of male insects, which have been sterilized by exposure to ionizing radiation, in sufficient numbers to out-compete fertile wild males for mating with wild females. Mating of sterile males with wild females reduces the number of viable progeny, resulting in a substantial decline in the overall size of the targeted local pest population (Bourtzis and Vreysen, 2021). Release of male insects has been found to be most cost-efficient in SIT programs (Lutrat et al., 2019), and generally raises fewer risk concerns. There exist accepted processes and protocols for SIT, including methods for *Aedes aegypti* mosquitoes (World Health Organization, 2020b; Dyck et al., 2021; International Atomic Energy Agency, 2023). Cage and small scale field trials of SIT for *Anopheles* species also have been initiated in Africa (Helinski et al., 2008; Republic of South Africa, 2018).

Another relevant precedent is Oxitec’s GM, but non-driving, OX5034 product for *Aedes aegypti* population suppression, which has been approved for commercial release in Brazil and is undergoing field testing in the United States (Government of Brazil, 2020; U.S. Environmental Protection Agency, 2020; Oxitec, 2023a). Rearing procedures for GM *Aedes aegypti* have been published (Carvalho et al., 2014). Other programs also are pursuing population suppression or population replacement strategies based on the release of live *Aedes* mosquitoes infected with *Wolbachia* bacteria (Zeng et al., 2022; Consolidated Mosquito Abatement District, 2023; MosquitoMate, 2023; National Environment Agency Singapore, 2023; World Mosquito Program, 2023).

### Identity

The standard of identity commonly describes the components a product must contain as well as those it may contain. In the manufacturing of GDMMs, methods will be required for routine authentication to confirm that the product retains the essential characteristics specified for the original regulatory approval for release to market (Benedict et al., 2018a). However, any identity standard for GDMMs must take into account inherent requirements of mosquito strain maintenance.

The practice of maintaining a well-characterized and protected master cell bank or seed stock is widely utilized in the manufacture of biological products to assure an ongoing supply of the originally characterized material, and cryopreservation is the method usually used for long-term storage (Hay, 1988; European Medicines Agency, 1998; U.S. Food and Drug Administration, 2010). Such a storage method is currently impractical for mosquitoes, however. Cryopreservation of mosquitoes generally has proven difficult due largely to characteristics of membrane permeability and chill sensitivity, and no reliable method for cryopreservation of any *An. gambiae* life stage is yet available (Gallichote et al., 2023). Cryopreservation remains an area of active research and a recent report of success with *An. stephensi* could hold promise (James et al., 2022).

The current inability to cryobank seed stock of *An. gambiae* GDMMs results in a need for transgenic lines to be continuously maintained through all life stages. This is not an unusual feature for insect products, and also is routinely the case for insects used in SIT. Mass production of GDMMs is expected to begin with establishment of a mother colony (U.S. Centers for Disease Control and Prevention, 2011; U.N. Food and Agriculture Organization, 2017). Maintenance of mosquito lines is more complex than some other insects in that it involves providing for both water-dwelling larval stages and blood-feeding adult stages. Colony failure at a production facility is a risk for any insect product, but may be particularly onerous for *Anopheles* GDMMs since the inability to cryobank seed stock could result in the need to *de novo* recreate the strain. It is not known how different regulatory authorities might choose to interpret the scientific equivalence of a *de novo* rederived line versus the initial line upon which approval of the product was based. The possibility that such an event might require new testing or new approval makes it prudent to maintain the strain at multiple sites to assure a dependable source of the GDMM stock in case of colony failure at one production facility.

Minor variations in the genetic background may be introduced in the course of protracted maintenance of the original GDMM strain as a result of the accumulation of random, or gene drive-system-induced, mutations as well as strain evolution in the laboratory or insectary. Avoidance of adaptation to insectary environments, potentially resulting in reduced fitness and diminished effectiveness, may necessitate occasional refreshing of the GDMM colony through crossing with wild-type mosquitoes. Although such refreshing introduces the possibility of changes in the genetic background of the GDMMs over time, this issue is common to all live insect products as well as other colony-managed animals. Indeed, needs for strain maintenance and replacement have been extensively addressed for SIT (Dyck et al., 2021). There also may be reasons to customize the GDMM product to local circumstances. For example, this could be desirable if the local vector population is substantially more insecticide resistant than the GDMM strain, which might put the GDMMs at a temporary disadvantage for establishment (Garcia et al., 2019). This possibility of local customization has likewise been suggested as a means to enhance mating compatibility with local mosquito populations or avoid inadvertently introducing new characteristics into the native population. If required, such customization could be done by introduction, through repeated backcrossing with mosquitoes of the local genetic background. Alternatively, it could be accomplished by transformation of local mosquitoes using the transgenic construct; in this case, however, the ramifications for product approval must be kept in mind (Connolly et al., 2023). Whether a new transformation event would be more likely to be considered a new product than one derived by introduction or introgression, as well as the types of data and information required for decision-making in each of these cases, will be determined by regulatory authorities.

Since strain maintenance is likely to require ongoing interbreeding and possible occasional outbreeding, it has been recommended that GDMM authentication methods concentrate only on the most distinctive characteristics of the strain (Benedict et al., 2018a). Suggested identity criteria for GDMMs therefore focus on the description of the transgenic construct, including copy number and location in the mosquito genome, because precedent indicates that the transformation event is expected to be the regulated article and the expression product of the construct is responsible for the direct effect (James et al., 2023). For other GM animals, identity characterization also focuses largely on the transgenic construct and its stability across generations (U.S. Food and Drug Administration, 2015). The identity method should be sufficiently discriminatory to show that the construct remains as described in the application for approval. The lack of emphasis on details of the genetic background of the mosquito is consistent with the lack of a formal regulatory standard for identity of SIT products, where the norm is simply to use a local strain of the targeted pest species (J. Bouyer, personal communication) and to allow colony refresh through introduction of the wild type as necessary (Dyck et al., 2021).

### Potency

For live mosquito products, the potency standard will equate to performance characteristics. Estimates of performance during production must be based on selected surrogate indicators that can be routinely measured in the insectary and are considered reflective of the product’s ability to perform its claimed function. As an example, for SIT with *Ae. aegypti*, performance generally relates to fitness and behavioral characteristics, such as: “the male is capable of flying, surviving and dispersing in the environment; mixing with the wild population; competing with its wild counterparts in courting, mating with and inseminating wild females, thus reducing the probability of those females mating with fertile wild males” (World Health Organization, 2020b). However, assessment of some of these parameters requires highly involved and technically demanding field studies such as mark-release-recapture experiments (Benedict et al., 2018b) that are not amenable to routine operational application. Therefore, for SIT, measurement of emerging male mosquito flight capacity in insectary settings has been proposed as a useful performance test (Balestrino et al., 2017; Culbert et al., 2018; Culbert et al., 2020).

The most consequential surrogate indicators of performance may differ for different types of GDMM products. Parameters related to efficacy and safety of GM mosquitoes as described by the World Health Organization 2021 are likely possibilities from which to select key surrogate indicators on a case-specific basis. Proposed performance criteria for use in manufacturing include competitiveness of GDMM mosquitoes with regard to their wild counterparts, as well as strength of the gene drive construct, measured as spread through a population (James et al., 2023). Field testing will provide an important opportunity to evaluate proposed GDMM performance standards. Therefore, in planning for field trials, choice of indicators should take into consideration their future utility and cost as routine quality control surrogates for the final marketable product.

Setting minimal performance requirements for a live GDMM product will be more nuanced than setting potency standards for a chemical entity. For example, deficits in fitness can be overcome by release of increased numbers of GDMMs, deflecting the question of efficacy more toward cost and logistical issues. The extent to which such flexibility is allowable likely will depend upon the wording of the product claim. In the particular case of GDMMs for malaria control, the efficacy required of a viable product also may be dependent on the disease transmission level at the treatment site and therefore variable according to local conditions, including availability and effectiveness of other control methods. Pre-approval testing may need to explore these variables so that the label language can adequately represent the potential variety of product uses.

Generally, performance standards are set by manufacturers according to the product claim and use case. With SIT for other insects, for example, performance requirements regarding flight ability or survival are not imposed by regulators but are a matter of negotiation between the manufacturer and the user (J. Bouyer, personal communication). Likewise for GDMMs, performance standards would best be established by the manufacturer according to local conditions and user requirements. Regulators will, however, require data demonstrating that performance supports the product claim.

### Quality

For other live mosquito products, as well as GM crops, demonstration of the maintenance of product quality is the responsibility of the manufacturer (J. Bouyer, S. O’Neill, personal communication). Manufacturers will need to be proactive in establishing their own quality standards to protect the reputation of the product, and discussing these with the regulators. Considerations likely would include characteristics associated with ability to achieve the product claim, such as fitness and maintenance of the transgenic construct in a functional form, presence of any contaminating mosquito species, or, for products which involve male-only releases, percentage presence of female mosquitoes from the product line. Quality management systems will be needed at production facilities to ensure that the GDMM product consistently and reliably meets the applicable identity and performance requirements. While good manufacturing practice is not defined for GDMMs, certain common principles (Sarvari et al., 2020) that would be pertinent to GDMM production include: designing and constructing the facilities and equipment properly; writing sufficiently detailed, comprehensible, standard operating procedures (SOPs) and instructions, with updating as needed; confirming these processes; following written procedures and evaluating and maintaining records of staff performance and training; establishing a record-keeping system and documenting work; monitoring and regularly inspecting facilities and calibrating and maintaining equipment; ensuring the quality of materials and protecting against contamination; conducting planned and periodic audits that help to recognize any errors and correct noncompliance; and, promoting workplace quality and safety.

For SIT programs (Dyck et al., 2021), in addition to ongoing performance evaluation as discussed above, other quality considerations have been classified as: production control (defined as monitoring all aspects of insect rearing, including materials and equipment used, personnel and environment); and process control (defined as measuring how things are done to identify possible sources of variability). SIT programs, especially those developing methods for *Ae. aegypti* mosquitoes, can provide context for both production and process control. The specifics of facility design, colonization, rearing, handling and strain maintenance have been well described (Food and Agriculture Organization, 2012; International Atomic Energy Agency, 2017; Dyck et al., 2021). Certification of rearing facilities, as required by Good Laboratory Practices or ISO 9000 standards, has not generally been considered necessary, although this may vary by country. A need for external inspection, qualification and permitting of the production facility, e.g., by national authorities and/or WHO Prequalification Inspection Services, can be expected (James et al., 2018; World Health Organization, 2020b; Dyck et al., 2021; World Health Organization, 2023b).

Quality control considerations will include avoiding colony contamination by: verifying the species of any locally-derived mosquitoes introduced into the colony; ensuring the absence of human or animal disease agents known to be transmitted by the mosquito species; and, safe-guarding that appropriate containment procedures are in place to prevent cross-contamination among different GDMM strains (Benedict et al., 2018a; American Committee of Medical Entomology, 2022). Housing and feeding of both aquatic and adult life stages will require materials, such as larval water and food source and adult sugar and blood source, that meet applicable regulatory requirements.

Ability to detect non-conformance with agreed upon identity and performance expectations and to initiate appropriate corrective actions within the manufacturing process is an important component of quality management. Appropriate data management systems will be key, enabling assimilation and assessment of quality metrics over time and quick identification of characteristics that are out of specification so that rapid remedial action can be taken (e.g., International Atomic Energy Agency, 2018). Some form of regular auditing can help to ensure the ongoing identity, performance and quality of the GDMM strains. Reports from internal audits may be required by the regulator, but it is likely that the regulator also will perform occasional audits. A report of product failure in the field arising from post-implementation monitoring could trigger an audit.

## Market entry

The market for GDMMs has yet to be explored since no product has to date been put forward. As with other aspects of the GDMM development pathway, processes established for market entry of conventional public health products are unlikely to be entirely applicable (James et al., 2018). Thus, complementary or additional mechanisms that may need to be put in place for operationalizing GDMM products should be considered. This involves identifying the potential customers and understanding their interests. While not excluding other possible uses, it has generally been assumed that GDMMs largely will be used by disease control programs in the context of their vector management activities and therefore the most likely customers will be health-related government agencies at the national, provincial and/or local level. Consideration of potential business models should recognize that government agencies may wish to establish their own GDMM manufacturing and delivery capabilities, acquire the GDMM product to deliver themselves, or contract for the GDMM product and all activities necessary to deliver it. Revisiting the SIT precedent, options may exist for both government-run programs and private suppliers, or a mix of both (Dyck et al., 2021). Early analysis of the market will support development of a product or service that is relevant to customer needs and help to clarify appropriate business models. It also will facilitate understanding among potential users of GDMMs as a new and possibly valuable tool for meeting their public health goals.

### Production facilities

Procedures for establishing SIT production facilities can provide useful guidance for scale-up production of GDMMs (Dyck et al., 2021). These identify factors for determining optimal location of individual production facilities, such as logistical access, availability of necessary resources (e.g., power and water), generally enabling government requirements, local acceptance, and labor availability. Establishment of production facilities will require adequately designed and equipped manufacturing facilities, well trained staff, and SOPs for mosquito husbandry, quality management, and documentation. Optimization of each element of the rearing process will underpin efficient scale-up production and reliable delivery of high-quality products. Production of GDMMs may involve additional provisions for the biosafety for GMOs that are not ordinarily encountered by SIT programs (e.g., Australian Government, 2011; U.S. Food and Drug Administration, 2015; U.S. Centers for Disease Control and Prevention, 2020; World Health Organization, 2020c). While containment considerations for research on GDMMs have been addressed elsewhere (American Committee of Medical Entomology, 2022), the appropriate containment measures for post-investigational manufacturing and distribution will need to be determined by regulatory authorities. It is possible that a favorable decision for wide-scale implementation will be accompanied by more relaxed containment requirements during production and transport. As mentioned earlier, if different GDMM strains will be maintained within the same production facility, facility design must anticipate a need for appropriate segregation of the separate strains, as well as ongoing testing to ensure that no mixing has occurred and a remediation plan in the event that cross-contamination is detected (Benedict et al., 2018a).

Launching qualified production sites for even small-scale production of *An. gambiae* GDMMs in Africa could well be an intensive multiyear process (Guissou et al., 2022). Planning for where these facilities should be located, how they must be designed, how they will be funded, and what quality management systems must be put in place should be considered as early as possible, as this will be vital for reducing what could amount to substantial lag time between a decision to implement and the actual ability to implement.

### Facility location

Other types of products are often manufactured in centralized facilities to take advantage of economy of scale (increased efficiency based on access to well-characterized equipment and processes and well-trained staff). This allows for maximum control of critical factors influencing product quality (Medcalf, 2016). Based on the SIT example, manufacturing of live insect products within a centralized mass rearing facility also has the benefit of simplifying quality assurance and reducing cost of production. Both of these issues are important determinants of product uptake. The applicability of a centralized production model for *An. gambiae* GDMMs, however, depends on a number of issues encompassing both technical and political factors.

While similarly an issue for SIT and other live mosquito products, distance to the release area and limitations on shipping and delivery options pose particular complexities for *An. gambiae* GDMMs. *Anopheles gambiae* differ substantially from *Ae. aegypti*, which is the subject of most current work on live mosquito products. *Aedes aegypti* eggs maintain viability after extended periods of desiccation (Faull and Williams, 2015). This has allowed a centralized production model whereby eggs are shipped from a remote facility to field sites for short-term storage, production and distribution of other life stages, or even direct delivery to the field (Oxitec, 2023a; Oxitec, 2023b). However, the fact that cryopreservation is not yet reliable and that *An gambiae* eggs rapidly lose viability, even at low temperatures (Ebrahimi et al., 2014; Mazigo et al., 2019), may limit the prospects for routine long-range supply in quantities required for implementation. Continued research may identify ways to overcome these limitations in the future. For example, certain compacting and chilling conditions have allowed for short-term transport of adult male *An. arabiensis* up to 24 h (Culbert et al., 2017). However, at present, this limitation may dictate distance of the production facility from the release sites to allow for delivery of viable GDMMs with the necessary performance characteristics. This concern should be clarified during pre-approval testing, and simple assays for performance of transported GDMMs upon arrival at field sites should be determined at that time.

A distributed manufacturing model could help to address these delivery limitations. In the case of GDMMs for control of malaria in Africa, a distributed model could also support more local autonomy and entrepreneurial opportunities that would be attractive to government and public end users. This might take the form of regional, national, or even local production sites. Challenges associated with a highly distributed model include assuring that all individual production facilities meet applicable regulatory requirements, and that their GDMM products meet quality requirements and are equivalent functionally. In one possible version of the distributed model, transgenic mosquitos might be produced *de novo* in a facility near the release area by injection of the transgene DNA into local mosquitoes. As mentioned above however, this could have important regulatory implications concerning whether each new transformation event conducted in a different facility would be considered a new product (Connolly, 2023; James et al., 2023). Regional production could provide some of the advantages of a centralized model while still reducing distance between manufacturing and release sites.

Any form of centralized or regional production likely would benefit from agreement among involved countries to allow a GDMM product manufactured elsewhere to be introduced into their country (James et al., 2023). This might require market entry plans to be broached with potentially involved countries early in the development process to foster understanding of the expected benefits, identify concerns, and understand how any importation issues can be addressed. An international framework to facilitate transboundary shipments of sterile insects has been proposed (Enkerlin and Pereira, 2022), and mechanisms for harmonization of regulatory requirements for GDMMs are being explored by the African Union (African Union Development Agency-NEPAD, 2023).

### Production processes

For a particular GDMM product, the scale of production required for implementation will be determined by the desired public health effect, which dictates a release plan to achieve the expected reduction in vector numbers and/or disease transmission over a specified area, perhaps within a specified timeframe. Although the scale of production required for self-limiting or localizing GDMMs is generally expected to be greater than for those that are self-sustaining, the more extensive the releases of self-sustaining GDMMS are the more quickly positive epidemiologic results can be expected. For both self-sustaining and self-limiting GDMMs, there may be limitations on the timing of releases with respect to seasonality that could require periodic surges in production. The range of anticipated release numbers, pattern and frequency of GDMM releases necessary to achieve the desired effect should be clarified via pre-approval field studies, although these may need to be further adjusted to meet the real world challenges of operationalizing the technology for implementation at scale.

Large-scale production of GDMMs will benefit from mechanization, which could help both to maintain quality and create a more affordable product. For other biotechnology products using a distributed production model, automation has been proposed as an important contributor to ensuring consistent product quality across different manufacturing locations (Medcalf, 2016). Additionally, for GDMMs designed for population suppression in which no means is available to suppress transgene effector expression and maintain the strain in homozygous form, complex rearing procedures may be necessary that involve backcrossing each generation with non-transgenic mosquitoes to maintain the transgenic element with screening and segregation by sex and transgene status at each generation. Mechanization of these highly labor-intensive steps would facilitate scaling of GDMMs for operational use even if release numbers are substantially lower than for conventional SIT approaches.

Depending on the insect species being produced for SIT, some level of mechanization has been achieved in almost all stages of the rearing process. Because of their aquatic stage, mosquito rearing is more complex and labor-intensive than rearing of certain other insects targeted for SIT. Studies on mass rearing of *An. arabiensis* underway in Sudan and South Africa (Maiga et al., 2020) and of *An. gambiae* s.l. in West Africa (Zubair et al., 2021) are yielding insights into mass rearing requirements for anophelines. Oxitec also is working to develop its GM Friendly™ mosquito technology in *An. stephensi* and *An. albimanus,* although this work is at an early stage (Oxitec, 2023c). While more advanced, mass production methods for *Aedes* currently still are adequate only at a relatively limited geographic scale (e.g., city-wide). However, sophisticated automation processes employing robotics for mass rearing are being tested (Crawford et al., 2020).

It has generally been assumed that releases of GDMMs into the field will consist of males, which would minimize any nuisance or risk posed by the possibility that released females could bite humans. As mentioned above, this precedent has been set by self-limiting genetic biocontrol approaches aimed at population suppression, in which large numbers of mosquitoes must be released on a continuous basis. With population replacement drives where the potential for disease transmission is greatly reduced and/or self-sustaining gene drives in which only low numbers of mosquitoes will be released, it is possible that any risks related to release of females will be judged acceptable. Precedent for mixed sex releases exists with the *Wolbachia*-mediated population replacement technology in *Aedes aegypti* (World Mosquito Program, 2023). If the intention is to release only male GDMMs, more facile methods to separate the sexes also could increase efficiency and reduce production costs. A variety of techniques have been used to separate male from female mosquitoes (Lutrat et al., 2019). For *Aedes* species, it is common to separate the sexes at the pupal or adult stages based on morphology, typically using sieves and/or plate separators (Carvalho et al., 2014), which is a labor and cost intensive process and can result in a small percentage of females remaining in the release batches. Recently, an automated process has been developed that separates out females based first on pupal body size and then by visual recognition of adult body parts (Crawford et al., 2020). Currently, pupal sex segregation methods developed for *Aedes* are unsuitable for *An. gambiae*, where there are not distinct differences in size between male and female pupae. Sex separation remains an obstacle to scaling up of *Anopheles* GDMMs as current methods rely on individual sorting at pupae or adult stage by trained technicians. A sex-specific transgenic fluorescent marker has been used successfully to separate male from female larval stages by flow cytometry (e.g., Cateruccia et al., 2005; Marois et al., 2012) and efforts are underway to make this method amenable to field use. It may eventually be possible to adapt some aspects of the automated sorting systems based on image recognition of adults developed for *Aedes* (Crawford et al., 2020; Senecio, 2023a) to *Anopheles*, but the sophisticated equipment required may be difficult to obtain and maintain in a developing country setting and/or, for a distributed manufacturing model, in multiple locations. Other alternatives for sex sorting also are being explored (Lutrat et al., 2019). These include spiking the blood meal with mosquito toxicants to kill blood feeding females (Yamada et al., 2013; Gunathilaka et al., 2019), using RNAi-based sex distorter systems to deplete females (Hoang et al., 2016; Taracena et al., 2019), as well as genetic sexing mechanisms (Meza et al., 2018; Mysore et al., 2021; Spinner et al., 2022).

### Distribution and delivery

Plans for market entry of GDMMs as a malaria control tool will need to include efficient methods for achieving the necessary level of area-wide coverage. Programs utilizing GDMMs to prevent malaria in Africa may have goals that range in ambition from control of transmission in an urban/peri-urban area (Doumbe-Belisse et al., 2021; Tadesse et al., 2021; World Health Organization, 2022b) to reduction of transmission in largely rural regions (World Health Organization, 2015; World Health Organization, 2020d; World Health Organization, 2022a) to malaria eradication across the continent (Feachem et al., 2019; World Health Organization, 2019). These different goals translate to vastly different coverage requirements. Depending upon the location of production facilities, it may be possible to perform releases directly or there may be a need for some form of intermediary staging facility. For example, some SIT programs have established a model of centralized production combined with local emergence and release facilities (Dyck et al., 2021). In this case, distribution of GDMM products would be a two-step process that involves distribution from a centralized or regional production facility to local staging facilities followed by delivery to more widely dispersed release sites.

In any situation where the production facility is remote from the release sites, protocols for transportation of GDMMs, such as storage conditions, temperature monitoring, tracking, labelling, disposition of shipping materials, and record keeping, will need to be prepared and tested in advance (e.g., World Organization for Animal Health, 2022; IATA, 2023). For centralized or regional facilities, involving transport across national boundaries, aspects of international shipping of live mosquito products include regulatory permit and health inspection requirements, containment and chain of custody issues, and challenges of ensuring product integrity/quality during handling. Certain of these requirements may differ according to the life stage that is shipped/transported. International guidelines have been developed for transboundary shipment of irradiated sterile insects (U.N. Food and Agriculture Organization, 2022) and for biological control agents more broadly (U.N. Food and Agriculture Organization, 2005). Gene drive modifications may require the imposition of additional conditions for shipping, not only to provide a level of containment necessary to avoid inadvertent release in transit that might result in unauthorized establishment of the mosquito but also to satisfy notification requirements for transboundary movement of GMOs if applicable Clearing House, Biosafety (2023). Conditions of the shipping route, i.e., how many stops are involved, how much time is required for transit, possibility of seasonal temperature effects, and regulatory requirements of transit countries, will be an important consideration for location of the manufacturing facility. Any particular requirements or restrictions on international transport of GDMMs should be explored before decisions about facility location(s) are reached.

More work is needed to develop efficient mechanisms for delivery of GDMMs to widespread release sites. Agricultural SIT programs have identified three basic mechanisms for insect release: ground-based containers, mobile ground-based vehicles, and aerial vehicles. The advantages and disadvantages of each approach have been extensively described (Dyck et al., 2021). To summarize, placement of ground-based receptacles containing pupae or eggs is labor intensive and subject to limited access, weather, human and animal intervention, and predation. Mobile ground release of adults, for example, from trucks or vans, requires fewer workers and can treat a larger though still limited area, but distribution remains subject to access (roads and terrain, weather). The expected ability of gene drive modifications to spread beyond the site of release may, however, reduce the challenges of limited access with ground-based approaches that has been experienced with other live mosquito products. Aerial release of adults can cover larger areas regardless of terrain, with the distance as well as quantity and viability of the payload being determined by the type of vehicle (e.g., fixed wing or rotary aircraft, various types of unmanned aerial vehicles (UAVs)), the packaging, and the release mechanism. However, aerial delivery can require expensive equipment and trained operators, is subject to weather, and may impose survival or fitness costs.

To date, programs releasing living *Ae. aegypti* mosquitoes have aimed for coverage over limited areas (towns, cities or suburbs). This has most often involved collection of adults or eggs from a local production facility and same day delivery by van or truck to release sites. Release methods have included manual placement of egg-containing boxes at relatively protected sites or discharge of adults from various types of cartons or tubes in yards or streets. However, methods for longer-term transport of irradiated *Ae. aegypti* adults are improving, which may expand opportunities for SIT programs (Maiga et al., 2023). Limitations to the area that can be covered by these manual methods have led to the exploration of alternative mechanisms to facilitate broader access. Such ideas include the use of mini-mobile laboratories for production (Public Broadcasting System, 2018) and new concepts for automated ground release or releases from UAVs or fixed wing aircraft (e.g., Bouyer et al., 2020; Marina et al., 2022; Senecio, 2023b).

Release of *An. gambiae* eggs does not currently seem a feasible option, at least in rural areas, because of their limited viability as well as the abundance of predators expected under field conditions. Although chilling techniques have been adapted to adult mosquitoes that prolong their viability (Bailey et al., 1979; Culbert et al., 2017), the distance that potentially can be covered by transporting *An. gambiae* adults in ground-based vehicles to release sites is likely to be restricted even in the presence of accessible roads, which are not always a given. While aerial transport and release across large areas using fixed or rotary wing aircraft has become standard for SIT against certain insect pests, similar options generally have been limited by the inherent fragility of adult mosquitoes. As reported to date for mosquito release, UAV transport has been piloted within fairly limited areas (e.g., Francaise, 2021; Bouyer et al., 2020). Experimentation to expand options for UAV transport is being actively pursued, however (Mechan et al., 2023).

## Discussion

GDMMs are being proposed as new tools to control and eliminate malaria in Africa because currently available control methods thus far have proven insufficient to achieve global goals and disease incidence has recently shown signs of rebounding (World Health Organization, 2022a). GDMMs have a number of theoretical advantages for preventing malaria transmission, including their utility against difficult-to-reach mosquito populations, the equitability of their effect regardless of socioeconomic conditions, and their ability to function in situations where other control methods have been disrupted (World Health Organization, 2021). Promising results from caged testing of GDMMs thus far support the potential of these technologies (e.g., Kyrou et al., 2018; Carballar-Lejarazu et al., 2020; Hammond et al., 2021; Ellis et al., 2022). However, challenges are expected with operationalizing GDMMs for malaria control. Challenges relating to risk analysis, regulatory and policy frameworks, as well as ethical concerns, are topics of ongoing discussion, and work is actively underway to address them (e.g., World Health Organization, 2021; James et al., 2023). Planning for other aspects of implementation, including manufacturing and delivery requirements, has to date been less of a priority. Nonetheless, several issues remain to be addressed to prepare for eventual market entry of these new products and new or updated mechanisms may need to be put in place, which could require substantial planning, time, and coordination. The best fit for handling live mosquito products will have to be determined for each country or region where these products will be deployed.

A major challenge relates simply to raising awareness of GDMMs among regulators and other decision-makers in disease endemic countries. Although GDMMs are expected largely to be implemented for public health benefit in the context of disease control programs, they differ from conventional public health tools such as drugs, vaccines and insecticides, in important ways. GDMMs are classified as living modified organisms (Convention on Biological Diversity, 2017), and will be regulated for biosafety according to mechanisms described under the Cartagena Protocol for Biodiversity (Convention on Biological Diversity, 2000) in the 173 countries that are signatories to the Protocol. According to these mechanisms, multiple ministries are likely to participate in biosafety decision-making (James et al., 2023). Ministries of Health likely will be interested in the effectiveness of GDMMs for disease prevention as well as product safety. Health regulators may be most familiar with effectiveness criteria developed for conventional products that can be chemically or physiologically characterized and are intended for individual or household use. However, typical manufacturing standards focused on product identity, potency or quality will need to be adapted for GDMMs. This will require health regulators to become familiar with the characteristics of live mosquito products. For example, any identity requirement is best focused on the transgenic construct rather than the mosquito genetic background, since standard practices of mosquito husbandry are likely to introduce changes in the overall genetic makeup of the GDMM line over time. Setting a performance standard for a live GDMM product also will be less straightforward than setting potency standards for a chemical entity, since performance requirements will be influenced by the nature of the GDMM (e.g., spread and persistence), the release plan (e.g., size and frequency of GDMM releases), and local disease transmission conditions (e.g., size of the local vector population and use of other control measures). Thus, certain requirements will be best negotiated between the manufacturer and the user (presumably the national disease control program) rather than imposed by regulators, with the manufacturer being responsible for ensuring that these requirements are consistently met. Oversight of the product can be maintained through auditing during manufacturing as well as ongoing post-implementation efficacy monitoring. Regulatory processes applied for market entry of other whole organism products, such as agricultural SIT programs, GM crops and animals, or other modified mosquito products, will be informative in this regard.

Appropriate business models are only beginning to be explored. It is not too early to begin the necessary outreach to understand the potential market for GDMMs, as this information may shape some crucial decisions in the research and development pathway. The decision to incorporate any type of GDMMs into a national control program likely will be dependent upon perceived cost and differential advantage with respect to other malaria control tools that must be readministered with some degree of regularity (such as insecticide-treated nets, indoor residual spraying, chemotherapy, or the current RTS,S vaccine (World Health Organization, 2022a)). Sustainability is a critical issue for GDMM programs (Haakenstad et al., 2019; World Health Organization, 2022a). If GDMMs are able to provide more durable and low cost protection as predicted, this could result in a substantial advantage that should be attractive to national governments and other funders. Likewise, the value proposition will take into account the scale of the public health goal. The vast area of the malaria belt in Africa (Institute of Tropical Medicine Antwerp, 2022; World Bank, 2023) and the rural nature of much of this region could favor an *An. gambiae* GDMM designed to spread and persist to contribute to a malaria eradication goal (Feachem et al., 2019). More focal malaria control goals may be amenable to self-limiting and/or localizing products.

For other types of products, centralized production is known to provide cost advantages of economy of scale. Some *Ae. aegypti* live mosquito products are employing a centralized production approach, which is made possible because of the ability to ship their eggs over long distances. However, *An. gambiae* are known to be fragile in transport. Moreover, country regulations governing introduction of GM organisms within their respective boundaries and containment requirements related to the presence of driving transgenes may further complicate international distribution and delivery of GDMMs. These limitations, if not overcome, could favor a more distributed model with multiple production facilities in locations appropriate to attain the level of coverage necessary to achieve the public health goal. A distributed model may also offer advantages for local autonomy. Production requirements, and therefore the business model, also will be influenced by the nature of the GDMM product, whether self-sustaining, self-limiting or localizing. It is possible that the production scale required for implementation of low threshold self-sustaining GDMMs will not be large since the modification is intended to spread autonomously from small releases into interbreeding populations by mating. Self-limiting or localizing GDMM approaches, anticipated to require relatively more frequent or larger releases to maintain broad effectiveness, are expected to face greater challenges to the ability to produce GDMMs at the necessary scale. Production requirements also may have implications for quality control, which likely will be easier to maintain for ongoing versus intermittent manufacturing. Thus, while the possibility that self-sustaining GDMMs will need to be released in lower numbers and less frequently to maintain efficacy could translate to a production advantage, it also might raise the practical issue of how to maintain a robust infrastructure for only sporadic production. This issue might be addressed through a centralized approach, with sustained production of the GDMM product for multiple markets. In the distributed model, it could be addressed by manufacturing within the same facility of several types of GDMMs (for example, using the same construct in different *Anopheles* species) for alternating implementation campaigns.

Manufacturing efficiency and quality control could be enhanced by the further development of several enabling technologies. Mechanization of key production steps would benefit large-scale production and quality control of all types of GDMMs. Improvement of capabilities for mass rearing of *Anopheles*, including development of automation technologies that will be affordable and sustainable in developing countries, is an area ripe for further research. Other enabling activities that developers should consider during early research include methods for preservation of GDMM product seed stock and identification of high-throughput mechanisms for assessing product identity and performance that will be suitable for routine use in manufacturing and monitoring.

Release mechanisms currently in use offer a relatively limited area of coverage. This has been a hurdle for scale-up of other live mosquito products and likely will present a disadvantage for self-limiting GDMM approaches. Self-sustaining approaches may be substantially better able to overcome this challenge due to their ability to spread the modification by mating, but this will depend on the degree of connectedness of *An. gambiae* populations as well as the timeframe over which disease reduction is expected. Current coverage limitations for release of live mosquito products may be overcome by newer delivery possibilities, such as aerial mechanisms, if these can be made cost-effective.

Coordination with other vector control methods will be important for successful GDMM implementation. Delivery and release of GDMMs could be performed by staff of the national vector control program and/or other government programs or by their contracted agents. In this case, the timing of GDMM delivery with respect to insecticide-based vector control programs, such as indoor residual spraying, must be planned from the perspective of staff availability as well as to ensure that newly-released GDMMs are not depleted before the modification can become established within the local vector population, although this might be expected to have little effect on male mosquitoes relative to indoor-feeding females. While not specifically discussed here, post-release monitoring for safety and efficacy also will be an important aspect of GDMM implementation (World Health Organization, 2021). The more delivery and monitoring can be integrated with other activities routinely conducted by national disease control programs, the lower the additional effort and cost that might be expected for GDMM implementation.

Here we have considered some current practical challenges related to market entry for GDMMs, with a focus on those under development for malaria control in Africa (Box 2). Some of these activities are broadly applicable to all GDMM types, some are more relevant for one type of GDMM than another, and others will be specific to a particular GDMM product. While only a beginning, it is hoped that this initial analysis will focus attention on currently unresolved issues that are important for the ultimate success of GDMM products, and stimulate further planning and investment to address these issues. Those presently engaged in more upstream research on GDMMs may consider these analyses premature, yet beginning to tackle them now could help them avoid some costly mistakes later in the development pathway.

BOX 2 **Enabling activities for market entry of new GDMM products for malaria**

• Increased understanding of GDMM technologies among regulators and other relevant government authorities
• Market analysis and clarification of the value proposition for each product
• Development of the business model for each product
• Clarification of regulatory approval requirements and new product approval
• Identification of indicators to be used for quality management in manufacturing each product
• Improved methods for long-term storage and preservation of mosquito strains
• Improved methods for mechanization of rearing and sex separation processes that are both high-throughput and suitable for use in developing countries
• Development of efficient mechanisms for transportation and delivery to release sites
• Identification of requirements for integration of GDMMs with other malaria control methods
